# Modeling the Producibility of 3D Printing in Polylactic Acid Using Artificial Neural Networks and Fused Filament Fabrication

**DOI:** 10.3390/polym13193219

**Published:** 2021-09-23

**Authors:** Mohammad Saleh Meiabadi, Mahmoud Moradi, Mojtaba Karamimoghadam, Sina Ardabili, Mahdi Bodaghi, Manouchehr Shokri, Amir H. Mosavi

**Affiliations:** 1 Department of Mechanical Engineering, École de Technologie Supérieure, Montreal, QC H3C 1K3, Canada; mohammadsaleh.sheikhmohammadmeiabadi.1@ens.etsmtl.ca; 2 Faculty of Engineering, Environment and Computing, School of Mechanical, Aerospace and Automotive Engineering, Coventry University, Coventry CV1 2JH, UK; mojtaba.kmoghadam1991@gmail.com; 3 Department of Biosystem Engineering, University of Mohaghegh Ardabili, Ardabil 5619911367, Iran; s.ardabili@ieee.org; 4 Department of Engineering, School of Science and Technology, Nottingham Trent University, Nottingham NG11 8NS, UK; Mahdi.bodaghi@ntu.ac.uk; 5 Institute of Structural Mechanics, Bauhaus-Universität Weimar, 99423 Weimar, Germany; manouchehr.shokri@uni-weimar.de; 6 Institute of Software Design and Development, Obuda University, 1034 Budapest, Hungary; 7 Alexander von Humboldt-Stiftung, 53229 Bonn, Germany; 8 Institute of Information Society, University of Public Service, 1083 Budapest, Hungary

**Keywords:** fused filament fabrication, toughness, 3D printing, machine learning, deep learning, artificial intelligence, computational mechanics, materials design, big data, data science

## Abstract

Polylactic acid (PLA) is a highly applicable material that is used in 3D printers due to some significant features such as its deformation property and affordable cost. For improvement of the end-use quality, it is of significant importance to enhance the quality of fused filament fabrication (FFF)-printed objects in PLA. The purpose of this investigation was to boost toughness and to reduce the production cost of the FFF-printed tensile test samples with the desired part thickness. To remove the need for numerous and idle printing samples, the response surface method (RSM) was used. Statistical analysis was performed to deal with this concern by considering extruder temperature (ET), infill percentage (IP), and layer thickness (LT) as controlled factors. The artificial intelligence method of artificial neural network (ANN) and ANN-genetic algorithm (ANN-GA) were further developed to estimate the toughness, part thickness, and production-cost-dependent variables. Results were evaluated by correlation coefficient and RMSE values. According to the modeling results, ANN-GA as a hybrid machine learning (ML) technique could enhance the accuracy of modeling by about 7.5, 11.5, and 4.5% for toughness, part thickness, and production cost, respectively, in comparison with those for the single ANN method. On the other hand, the optimization results confirm that the optimized specimen is cost-effective and able to comparatively undergo deformation, which enables the usability of printed PLA objects.

## 1. Introduction

Advances in novel additive manufacturing (AM) technologies are of utmost importance due to their higher flexibility, minimization of material wastes, and reduced tooling requirements [1]. Some evidence is emerging, in a number of industries, of the replacement of traditional manufacturing (TM) with AM. FFF is one of the most applicable AM technologies used to fabricate plastic products. An FFF printer directly builds 3D parts from a 3D computer-aided design (CAD) model by fusing successive extruded layers of feedstock material together to produce components layer by layer. Several studies have evaluated the effect of fused filament fabrication (FFF) process parameters on the mechanical properties and dimensional tolerances of printed parts by the design of the experimental methods [2,3,4,5,6] and by evolutionary algorithm [7]. Qattawi et al. [1] checked the effects of processing criteria on the mechanical properties and dimensional accuracy using 18 printed samples. Ceretti et al. [8] statistically analyzed the implementation of the two types of the process criteria and the extrusion head on the dimensions of multi-layered PCL scaffolds and pores in the deposited material using a modified FFF printer. The extrusion heads were a wire extrusion and a powder extrusion head. Extrusion head type did not strongly influence the resulting geometry of the samples. Griffiths et al. [9] used the design of experiments (DOE) method to quantify the effects of build. The results indicated that infill percentage and number of shells are significant factors to optimize tensile properties. Moreover, the maximum layer thickness and lowest infill percentage as well as the number of shells have to be used to optimize efficiency outputs. Lieneke et al. [10] developed a method to identify realistic tolerance values for additive manufacturing and factors influencing the geometrical accuracy. The materials, machines, and process parameters for FFF, laser sintering (LS), and laser melting (LM) were defined for the development of the method. Rezaie et al.’s [11] objective was to study the implementation of a mathematical tool used in the conceptual design stage for topology optimization. They investigated the application of topology optimization for the production of meso-scale structures to realize intermediated density regions. Mahmood et al. [6] applied Taguchi’s experimental method to test the effects of process parameters on structural definitude and geometric characteristics [7].

The mechanical properties of additively manufactured parts suffer compared to conventionally manufactured parts [12]. PLA presents a relatively brittle behavior under tensile loading [13]. Although the dimensional accuracy and mechanical properties of PLA have been already studied, there is little literature on the printed PLA’s toughness with a honeycomb internal fill pattern [14,15,16,17,18]. Hence, the effects of extruder temperature (ET), infill percentage (IP), and layer thickness (LT) and their interactions on toughness, thickness, and production cost of the 3D printed specimens in PLA were investigated by response surface methodology (RSM). One reason that may be more significant when using RSM in many scientific studies is that this method better shows the interaction between parameters and by graphic diagrams [7]. Recently, machine learning (ML) techniques have become one of the most effective tools for modeling and simulating scientific phenomena, mechanical properties, engineering processes, and different material behaviors in mechanical engineering fields. This section presents the notable studies that have employed ML techniques to handle modeling and predicting tasks in FFF 3D printers. Buys et al. [14] conducted research on 3D printers for the multi-material structure of the polymeric matrix. In this research, they evaluated the mechanical properties of samples such as wear, flexural, and morphological properties. The PLA-PA6/TiO_2_ polymeric matrix was printed and the wear examination showed that the wear rate for the PA6/TiO_2_ samples was 823 µm and 1092 µm for the PLA samples. Yadav et al. [14] employed an adaptive neuro-fuzzy inference system (ANFIS) as a hybrid ML technique for the prediction of tensile strength in PETG and ABS in the presence of temperature, material density, and layer height as input variables. Results were evaluated by error percentage. According to the results, the ANFIS could successfully cope with the task by an error percentage of 2.63. The maximum tensile strength was estimated to be 0.0405 kN/mm^2^ for PETG in the presence of a 0.1 mm layer height, material density of 1.27 g/cm^3^, and extrusion temperature of 225 °C. Ali and Chowdary [15] employed ANN for the prediction of the mechanical characteristics of FFF printed parts in the presence of air gap, raster angle, number of contours, and build orientation as input variables. ANN was trained using a Bayesian function. Results were evaluated by accuracy. According to the results, ANN could successfully cope with the task by enhancing the accuracy by about 5%. Sheoran and Kumar [16] developed a comparative study for analyzing GA, the Taguchi method, gray relational, RSM, ANN, fractional factorial, and fuzzy logic for handling the FFF approach to enhance the structural specifics as well as printed sample quality. According to the results, hybrid ML techniques improved the accuracy and increased performance compared to the single ML techniques.

According to the literature, the ML techniques can be effective tools for modeling the FFF process [14,15,16,17]. In addition, hybrid ML techniques provided higher accuracy and performance compared with single ML techniques [15]. This made us move toward comparing ANN as the frequently used and simple ML technique with ANN-GA as the hybrid ML technique. Therefore, the objectives of the present work can be categorized into two main stages. The first step was to improve the mechanical behavior of the FFF printed PLA under tensile loading and reduce the production cost of the specimens. The second step was to estimate the toughness (N-mm), part thickness (mm), and production cost ($) in the presence of LT, LP, and ET using the ANN and ANN-GA techniques. The honeycomb internal fill pattern was applied to increase the printed samples’ ductility and decrease material use. The area under force–extension curve up to fracture was considered the toughness of the printed specimens. The part thickness was measured by a micrometer of 0.01 mm resolution made by Mitutoyo (Mitutoyo Company, Model 500–196–30 AOS Absolute Digimatic Caliper, Kawasaki, Japan). The production cost was calculated using a formula based on reasonable prices in the FFF 3D printing market. The acquired data were analyzed by Design-Expert V8 software via the response surface method. The independent factors were optimized and examined to affirm that the research method was viable.

## 2. Materials and Methods

### 2.1. Response Surface Methodology and Artificial Neural Network-Genetic Algorithm (ANN-GA)

RSM is based on applied mathematics and the statistical techniques to determine functional relationships between output responses that are affected by input factors [17]. RSM generates an empirical polynomial model of approximation for response surface over a factor region [18]. The smaller the region of interest, the better the approximation when all the independent factors are continuous and can be estimated and regulated for experimental studies. Thus, the response surface can be presented through Equation (1) [19].

Y = f(*x*_1_, *x*_2_, *x*_3_, ..., *x*_*k*_) (1)

where *k* is the number of independent factors. The approximation of its mathematical model is represented through the infinite strings of *x* Taylor. The quadratic polynomial function expressed in Equation (2) is implemented in RSM [12,13,20].

(2) $$y=\beta_{0}+\overset{k}{\underset{i=1}{\sum }}\beta_{i}x_{i}+\overset{k}{\underset{i=1}{\sum }}\beta_{ii}x_{i}^{2}+\underset{i}{\sum }\underset{j}{\sum }\beta_{ij}x_{i}x_{j}+\varepsilon$$

where *β*, *β_i_*, *β_ii_*, and *β_ij_* are the constant, linear coefficients, coefficients of quadratic, and interaction coefficients, respectively. Furthermore, ε represents the regression error.

Here, the input factors include extruder temperature, infill percentage layer, and thickness. As discussed by Moradi et al. [19], the data were obtained from an experimental analysis using Design-Expert V8 software. Table 1 shows three factors (i.e., the statistical analysis based on Central Composite Design (CCD), full replication of three agents, and five stages). Based on the previous research [19], each of the factors was set at the significant domain because at these higher and lower ranges, the 3D printer has proper efficiency. Toughness (N-mm), part thickness (mm), and production cost ($) were opted as output responses. The samples were printed by FFF printer model Sizan 3 (Sizan Company, Kashan, Iran).

The part thickness was measured by a micrometer of 0.01 mm resolution made by Mitutoyo. The production cost of the specimens was calculated by a formula obtained from the 3D printing market. The cost of the FFF process was evaluated according to Lieneke et al. [21] which calculated the welding cost production. Equation (3) offers the production cost of PLA printed parts in terms of build time and part weight [21]. The design matrix and experimental results are reported in Table 2 [22]. Design experts uses the statistical analysis for input data and in this software, the central point and suggestion plan are proposed to generate proper parameters.

*Production Cost* = 0.5 *Build* time (min) + 0.03 Part weight (gr) (3)

ANNs are considered as computational intelligence tools inspired by biological neural networks [7,23]. ANNs train to do tasks by considering the existing mapping of the dataset. The architecture of an ANN is based on the interconnected layers through nodes. The nodes or so called neurons and each connection transmits a signal from one neuron to other neurons; the connections are like the synapses in a biological brain [24].

**Figure 1:**
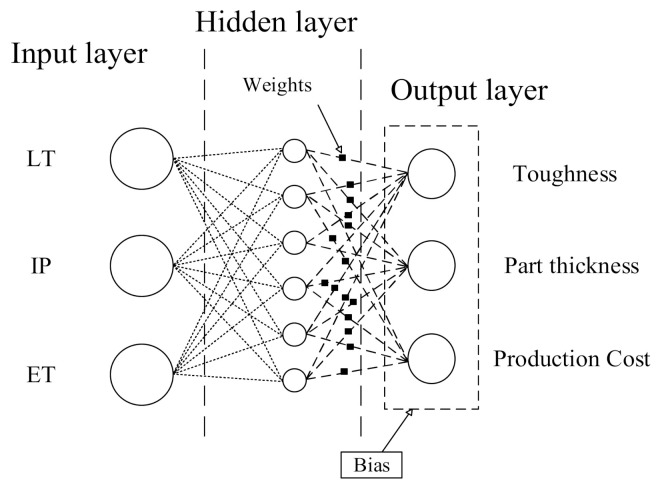
The architecture of an artificial neural network (ANN).

The output values of each neuron are affected by weight and bias values. All links between input layers and hidden layers compose the input weight matrix and all links between hidden layers and output layers compose the output weight matrix. Weight (*w*), which controls the propagation value (*x*) and the output value (*O*), from each node was modified using the value from the preceding layer according to Equation (4), which presents the relation for producing the output values of each neuron [25].

(4) $$O=f(T+\overset{n}{\underset{i=1}{\sum }}w_{i}x_{i})$$

where *T* is the specific threshold (bias) value for each node and *f* is a non-linear sigmoid function, which increases monotonically. The architecture of the proposed ANN is presented in Figure 1.

The training phase was performed by MATLAB software. For the implementation, LT, IP, and ET were used as input variables for the prediction of toughness, part thickness, and production cost, respectively. Seventy percent of total data were separated randomly for developing the training process by the network. The remaining data were employed for the testing process and to evaluate the accuracy of the network. The training phase was initiated using 10 neurons in the hidden layer and continued up to 16 neurons by intervals of two neurons. For each step, output data were generated and evaluated by the evaluation criteria in comparison with the target values.

**Figure 2:**
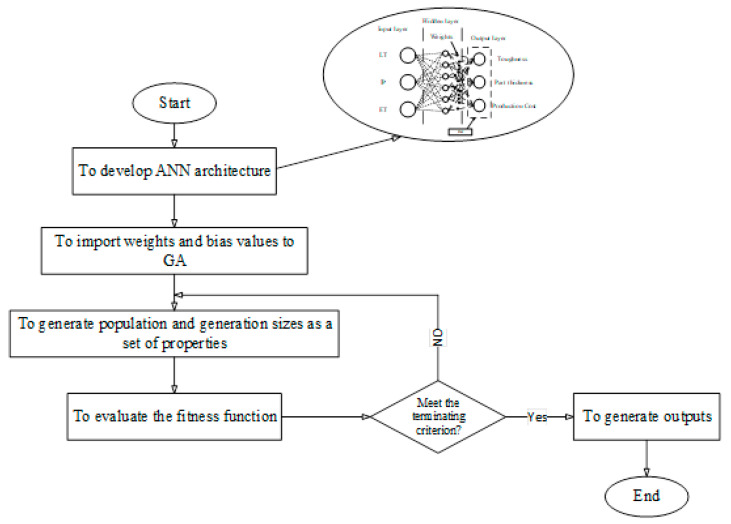
The flowchart of the artificial neural network-genetic algorithm (ANN-GA)-developing process.

Recently, hybrid methods have provided a higher accuracy compared to single techniques [26,27]. These techniques employ a predictor and an optimizer for developing an accurate prediction model. The general mechanism is to employ an optimizer for improving the architecture of the predictor to reach the best response. One of the frequently used and popular hybrid methods is ANN-GA. A population of candidate solutions to an optimization problem has evolved toward an optimal implementation in the GA. Each candidate solution has a set of properties to reduce the cost function errors. In the ANN-GA technique, the cost function is the output of layers as a function of weight and bias values. GA employs population and generation sizes as a set of properties and compounds as a cost function. The optimization of the cost function aims at reducing the error values. In this case, the error value reduction contributes to providing accurate outputs for the network compared to using a single ANN. Figure 2 represents the flowchart of the proposed machine learning hybrid model of ANN-GA, adapted, and reproduced from [28].

Table 3 presents the evaluation criteria that compared the predicted and output values. These factors are also called performance factors that handle the target and output values (the predicted by models). The correlation coefficient is an index to measure the linearity of the target and output values. The root mean square error calculates the deviation error of the output values compared to the target values [29]. These factors are considered the frequently used evaluation metrics in different modeling tasks [30].

### 2.2. Experimental Work

The tensile test samples fabricated in PLA were investigated mechanically, dimensionally, and economically. Polylactic acids are generated from renewable sources with numerous benefits and can be divided into categories, for example, PDLA (poly-D-lactic acid), PLLA (poly-L-lactic acid), and PDLLA (poly-DL-lactic acid) [31]. PLA has a low printing temperature and can be printed both with and without a heated print bed. The material properties of PLA are shown in Table 4, which was adapted from [31]. Despite all of these notable characteristics, PLA is brittle and it is not a true choice for items that might be bent, twisted, or dropped.

Simplify3D software was employed to fine-tune the build parameters of the specimens. Simplify3D includes comprehensive tools to work with 3D printers. The tensile test sample was modeled as a STL file by Solidworks (modeling computer-aided design and computer-aided engineering computer program, SolidWorks 2021 SP2.0, Dassault Systèmes, Concord, MA, USA) based on the international standard ISO 527–2 and imported into Simplify3D. Table 5 illustrates the definitions of the FFF build parameters that were permanent for all experiments.

**Figure 3:**
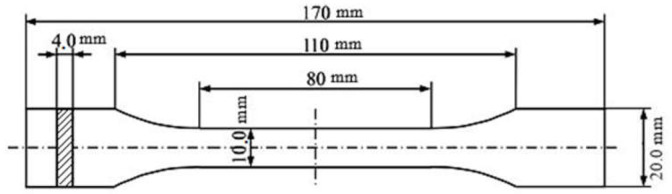
The sizes of the samples based on the ISO 527–2 standard for the tensile examination.

**Figure 4:**
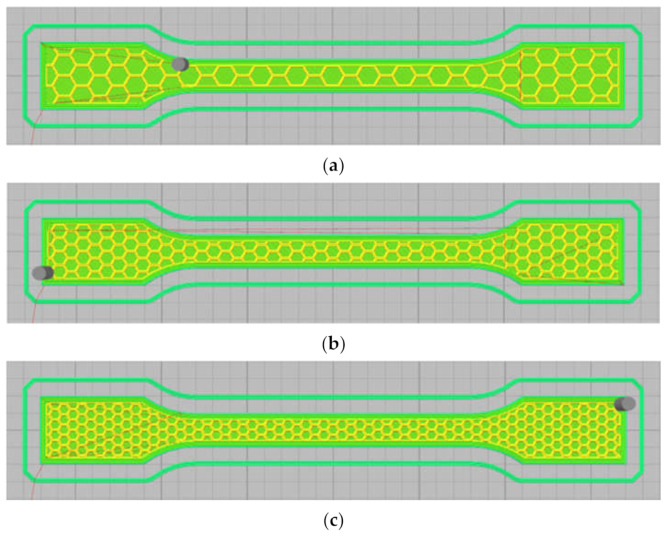
Honeycomb internal pattern at (**a**) 20%, (**b**) 30%, and (**c**) 40%.

**Figure 5:**
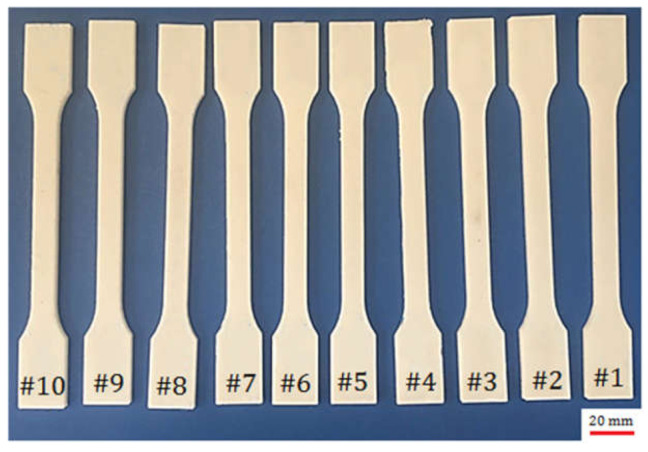
Fused filament fabrication (FFF) 3D printed parts of polylactic acid (PLA).

The infill pattern may significantly affect the strength of the 3D printed part. The honeycomb internal fill pattern was applied for the production of light-weight and high-strength specimens. The honeycomb internal fill adhered to the top and bottom solid surfaces offered an excellent rigidity. Figure 3 shows the sample size based on the ISO 527–2 standard for the tensile examination. Figure 4 depicts the 20%, 30%, and 40% full honeycomb infill. Figure 5 presents the 3D printed parts in PLA which is adapted from [24].

The SANTAM 150 universal test (SANTAM company, Tehran, Iran) was used to conduct tensile strength tests according to ASTM D638 at the constant rate of 2 mm/min. As Table 2 indicates, the specimens had two types of fracture under in-plane loading. Most of the specimens demonstrated brittle behavior with no visible deformation before fracture. Only five specimens showed a tough fracture and apparent deformation occurred before separation. These specimens had both a higher strength and ductility than that of the brittle specimens.

## 3. Results

The effects of the input factors on the outputs can be signified by the analysis of variance (ANOVA) results. The ANOVA is created by assuming that the elements are fixed, not random, and the design is crossed, not nested. The software selects polynomial terms in the mathematical model. However, the terms must be significant to refrain from aliasing of the model. Design-Expert calculates statistics such as the *P*-values, lack of fit, Adj R-Squared, and Pred R-Square values to appraise the models. The difference between the predicted adjusted R-squared and R-squared indicates whether the model can reliably be used to interpolate data. If the difference is less than 0.2, then the model fits the data and can be used to interpolate the data.

### 3.1. Toughness

**Figure 6:**
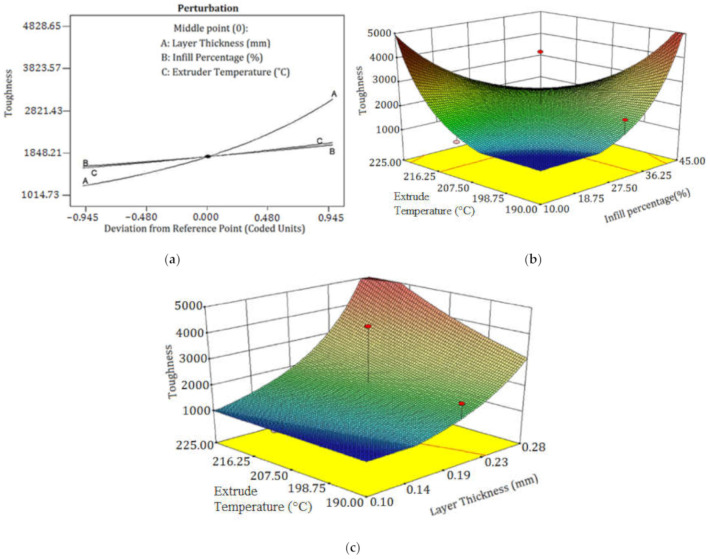
(**a**) Perturbation plot of toughness, (**b**) 3D-SP of toughness in terms of the extruder temperature (ET) and infill percentage (IP), and (**c**) 3D-SP of toughness in phrases of layer thickness (LT) and ET.

The analysis of the variance table showed that LT was the main controlling factor influencing toughness. The amount of *P*-value in this parameter was very low. Due to statistical analysis, when the *P*-value goes to the lowest amount, the parameter may has more effective. Interaction between infill percentage and extruder temperature is also crucial because when two parameters are considered at the same time, it is more tangible which one plays the central role. In Figure 6a and Table 6, the interaction of these parameters is shown. For toughness, the interaction between IP and ET was effective because the *P*-value had been placed in the effective range. Table 6 depicts the ANOVA results of toughness. The difference between predicted R-squared and adjusted R-squared was 0.042, which affirms that the model can efficiently interpolate data.

Equation (5) is the predictive model of toughness in terms of coded factors:

(*Toughness*)*^−0.41^* = +*0.045* − *8.760* × *10^−3^* LT − *2.795* × *10^−3^* IP − *2.369* × *10^−3^* ET + *5.605* × *10^−3^* (IP)(ET) (5)

Equation (6) is the predictive model of toughness in terms of actual values:

(*Toughness*)^*−0.41*^ = +*0.49164* − *0.17521* LT − *10.012049* IP − *01.91832* × *10^−3^* ET + *5.60471* × *10^−5^* (IP)(ET) (6)

**Figure 7:**
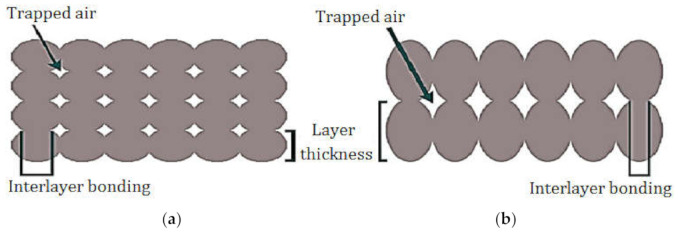
Interlayer bonding and trapped air using (**a**) lower LT and (**b**) higher LT.

**Figure 8:**
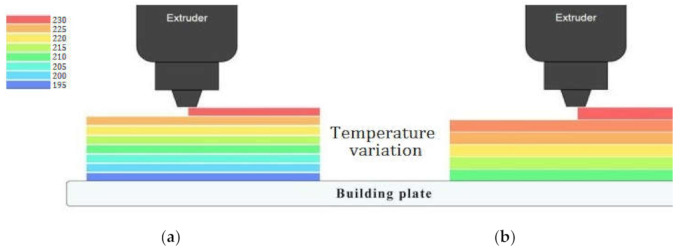
Temperature variation in (**a**) lower LT and (**b**) higher LT.

**Figure 9:**
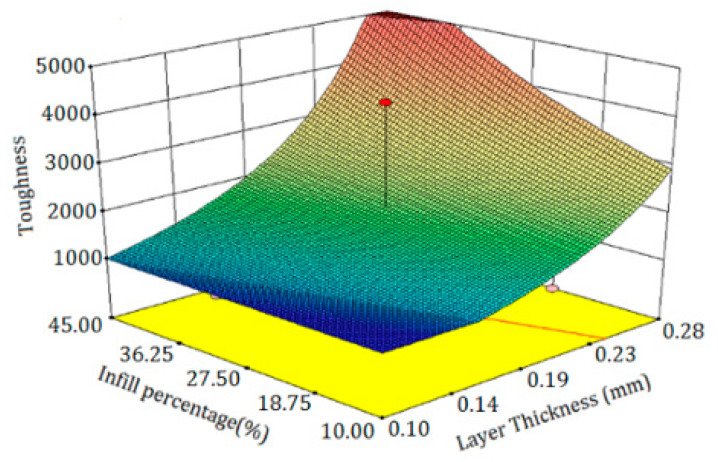
3D-SP of toughness in terms of IP and LT.

The relative significance of the factors can be obtained by comparing the coefficients of the factors. Figure 6a depicts the perturbation plot of toughness. A, B, and C curves illustrate the sensitivity of toughness to LT, IP, and ET, respectively. The plot indicates that the toughness of specimens was much more sensitive to LT than other controlled factors. The remarkable point is that IP and ET had a similar influence on the toughness while changing one factor and keeping the others constant. Figure 6b demonstrates the 3D surface plot of toughness in terms of ET and IP. The tough behavior in the printed PLA can be achieved by two procedures. The first is to increase the extruder temperature and decrease IP at the same time. The other is to increase IP and to decrease ET concurrently. The plausible arguments for the improvement in the toughness by the first procedure are the enhancement of interlayer adhesion between plastic strings at higher temperature and the reduction of the trapped air pockets between the strings at lower IP. Moreover, the time required to build the inside sections is considerably dependent on the IP. By increasing IP, the nozzle extrudes more hexagonal pattern lines at the inside sections, which takes more time considering the same printing speed for all cases of IP. Therefore, there is less time for heat transfer and variation in LTs using lower IP, which results in better fusion between plastic strings. Figure 6c depicts the 3D surface plot of toughness in terms of LT and ET. The surface plot indicates that increasing LT and ET at a time results in increasing toughness. In a specimen with higher LT, a smaller number of sections are needed to print the part. Therefore, a specimen with a thicker layer consists of less interlayer bonding, which are potential places to raise stress concentration and crack propagation. Figure 7 is beneficial to compare the interlayer bonding and trapped air using thin and thick LT. In addition, higher LT results in lower heat transfer rates and variation in layer temperatures [24] and consequently, better fusion and adhesion of the extruded layers on the solid layers is expected. Figure 8 demonstrates a schematic of temperature variation in lower and higher LT at the same printing speed. It is evident that printing PLA at lower temperatures results in poor layer bonding. The 3D surface plot (3D-SP) of toughness in terms of IP and LT is presented in Figure 9.

The 3D-SP implies that increasing LT and IP leads to an increase in toughness. The IP patterns and IP influence the interior solidity of the printed parts. For uniform stress distribution during the tensile test, hexagonal cells can withstand a mechanical load to impede stress increases on the neighboring cell. Additionally, IP specifies hexagonal cell size, and smaller cell sizes result in higher densities and higher strength. Therefore, it is rational to conceive that higher internal IP results in higher toughness.

### 3.2. Part Thickness

The dimensional accuracy of plastic printed parts is affected by many parameters. The melted strings are deposited based on the sliced G-Code file. In addition to the investigated parameters, the part geometry and printing speed may influence the strings’ placement and, thus, dimensional accuracy of the part. After it has been deposited out of the nozzle, the plastic behavior depends on plastic temperature, stable temperature, and ambient temperature. Although PLA does not shrink that much, it is essential to study the effects of process parameters on the dimensional accuracy of the printed parts in PLA. The variance table analysis indicates that although all input factors and their interactions influence the part thickness, the interaction between LT and IP is the significant parameter influencing the part thickness. In thickness, all parameters have an effective interaction. For example, by considering the interaction between LT and IP, the *P*-value is in range and the amount of this criterion is not high, so it leads to having an effective role. For other interactions such as LT, ET, and IP and ET, the *P*-value is in range, but the amount of the *P*-value in these two interactions is higher than LT and IP. Table 7 demonstrates the ANOVA results of the part thickness.

Equation (7) represents the anticipating part’s model thickness in terms of coded factors as follows.

*Thickness* = +*3.99* − *0.11* LT − *0.039* (IP) + *0.096* (ET) + *0.21* (LT)(IP) − *0.087* (LT)(ET) − *011* (IP)(ET) (7)

Equation (8) represents the anticipating part’s model thickness in terms of actual values:

*Thickness* = −*9.04550* + *21.77500* (LT) + *0.13688* (IP) + *0.076875* (ET) + *0.42500* (LT)(IP) − *0.17500* (LT)(ET) − *1.07500* × *10^−3^* (IP)(ET) (8)

**Figure 10:**
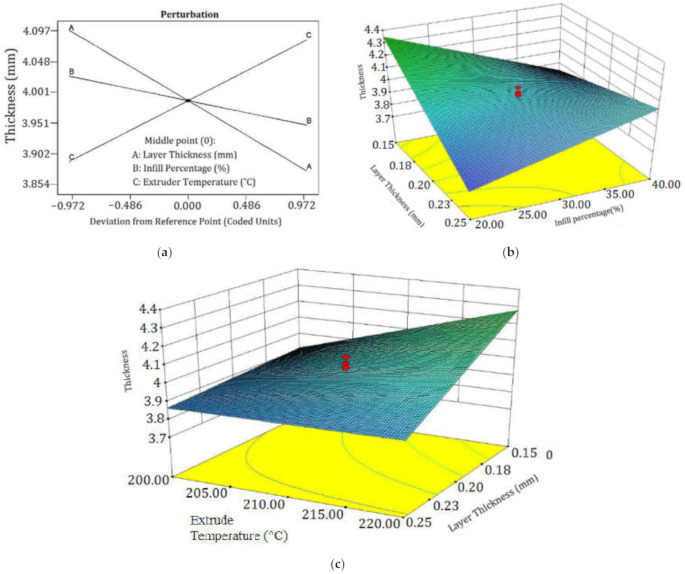
(**a**) Perturbation plot of thickness, (**b**) 3D-SP of thickness in phrases of LT and IP, and (**c**) 3D-SP of thickness in phrases of ET and LT.

Figure 10a shows a perturbation plot of the part thickness. The plot shows that part thickness is very sensitive to change in all controlled factors. It can also be observed that the central point of controlled factors (LT = 0.2 mm, IP = 30%, and ET = 210 °C) is a suitable setting to reach the desired part thickness. Figure 10b depicts the 3D surface plot (3D-SP) of part thickness in terms of LT and IP. The 3D-SP of part thickness in phases of ET and LT is presented in Figure 10c. In Figure 10b, by increasing IP the thickness has increased and by decreasing LT, the thickness has decreased. Also in Figure 10c, the thickness has decreased by LT, but the ET may not be very effective to change the thickness.

### 3.3. Production Cost

The ANOVA illustrates that the LT and IP are the most important factor influences the production cost. LT and IP have a proper *P*-value and their amount is <0.0001. Table 8 depicts the ANOVA outputs of production cost. Additionally, “Adj R-squared” and “Pred R-squared” were in excellent agreement.

Equation (9) expresses the anticipating model of production cost in terms of coded factors:

(*Production Cost*)^*−1.68*^ = +*7965* × *10^−3^* + *2.030* × *10^−3^* (LT) − *3.117* × *10^−4^* (IP) + *4.329* × *10^−5^* (IP)*^2^* + *8.552* × *10^−5^* (ET)*^2^* (9)

Equation (10) expresses the anticipating model of production cost in terms of actual values:

(*Production Cost*)^−*1.68*^ = +*0.038884* + *0.040595* (LT) − *5.71489* × *10^−5^* (IP) − *3.59174* × *10^−4^* (ET) + *4.32947* × *10^−7^* (IP)^*2*^ + *8.55175* × *10^−7^* (ET)^*2*^ (10)

**Figure 11:**
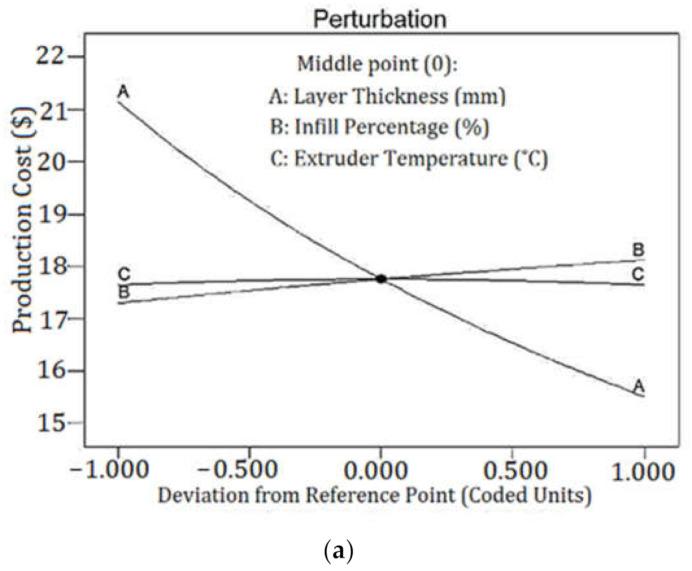

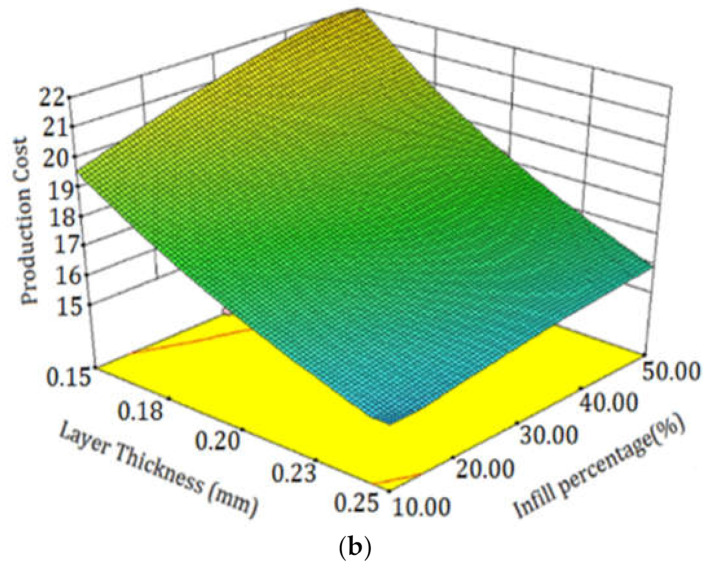
(**a**) Perturbation plot of production cost and (**b**) 3D-SP of production cost in phases of LT and IP.

As the coded equation shows, LT had the highest coefficients among the equation terms. Figure 11a depicts a perturbation plot of production cost. The plot confirmed that production cost was much more sensitive to LT than other input parameters. Figure 11b shows the effects of LT and IP on the production cost in the form of a 3D surface. Build time had a major impact on the production cost based on the suggested equation. The build time is the sum of the extruding time of top and bottom solid surfaces and inside sections. The parameter that defines the number of sections to produce a part is LT, and the parameter that determines the extruding time of the inside sections is IP.

### 3.4. ANN and ANN-GA Techniques

ANN and ANN-GA techniques were performed to develop an accurate model for the prediction of toughness, part thickness, and production cost. In the first step, an ANN was developed by 10, 12, 14, and 16 neurons in its single hidden layer for choosing the best number of neurons in the hidden layer in the presence of 70% of the total data. Results were evaluated by correlation coefficient and RMSE values and were tabulated in Table 9. The best response was related to neuron number 12, with the values of 734.6853877 and 0.8692 for RMSE and correlation coefficient, respectively. Therefore, the architecture of 3–12–3 was selected as the base ANN architecture to be optimized by GA. In the Table 9. The following abbreviations, the Pop. Size, Max Gen., and the No. of Neurons stand for population size, maximum generation, and number of neurons, simultaneously.

GA implemented the ANN’s selected architecture in four treatments (based on our experiences in previous studies). These treatments included a population size of 50, 100, 150, and 200. The results are tabulated in Table 9. Based on Table 9, a population size of 150 with a maximum generation size of 360 provided higher accuracy for toughness and production cost and a population size of 100 for part thickness compared with other population sizes. This population size increased the accuracy by about 9.7%, 5.8%, and 1.2%, respectively, for toughness, part thickness, and production cost compared with a single ANN.

By considering the training stage, the elected architectures were employed for the testing stage. The results are tabulated in Table 10. As is clear, the accuracy of the testing and training stage for single ANN did not match, in other words, there was a larger difference between the accuracy of the testing and training stages for the single ANN method. This makes ANN an untrusted approach. On the other hand, hybrid ANN-GA benefits higher sustainability by comparing the testing and training results, which provided almost similar accuracy.

**Figure 12:**
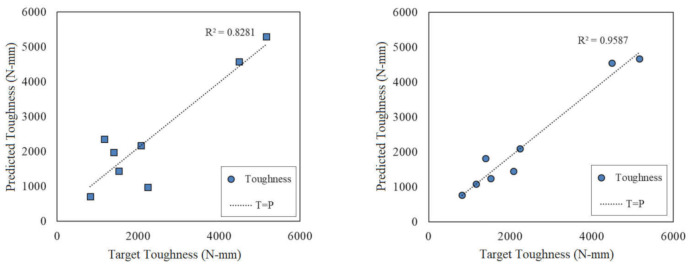

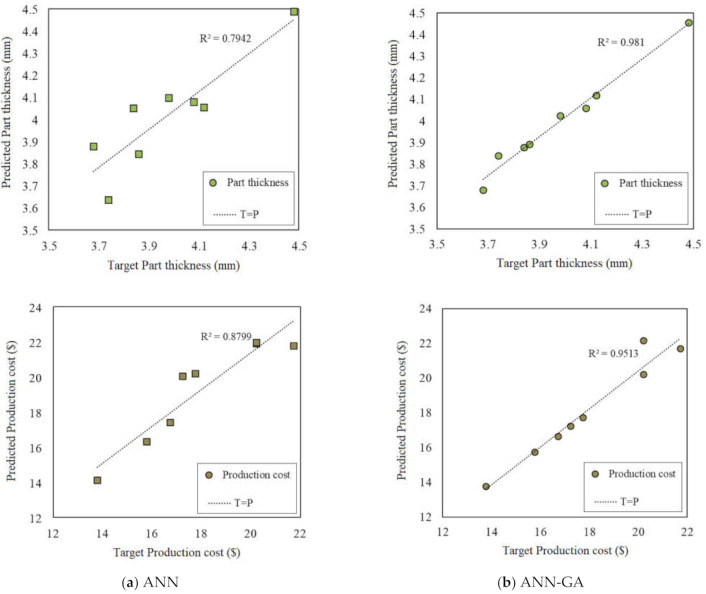
Plot diagrams for the testing phase. (**a**) Single ANN-GA and (**b**) hybrid ANN.

Figure 12 presents the plot diagrams for ANN and ANN-GA in the testing stage. This plot presents the predicted values on the vertical axis and target values on the horizontal axis. Line T = P is the reference one-by-one line to determine the correlation values. Deviation from this line indicated the error value between the target and predicted values. Based on Figure 12, ANN-GA provided a higher correlation for the target and expected values compared with those of the single ANN method. The part thickness and production costs were due to the higher accuracy of the ANN-GA compared with that for the toughness.

**Figure 13:**
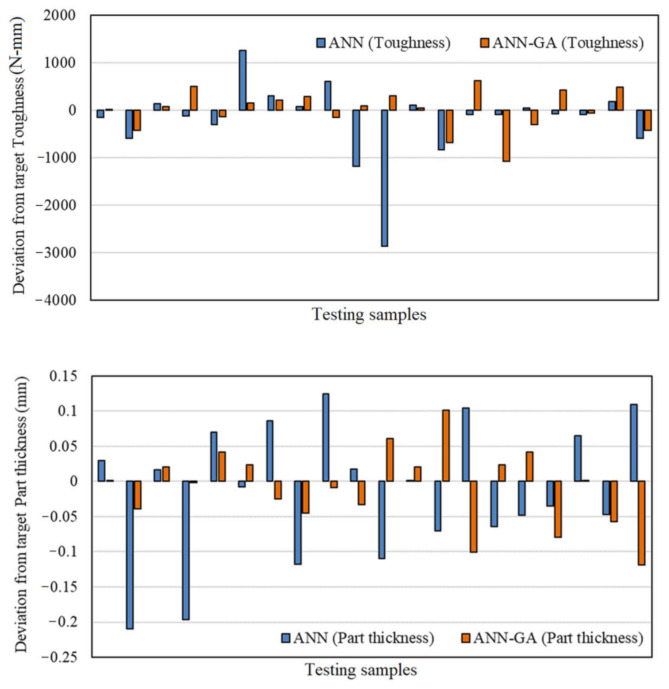

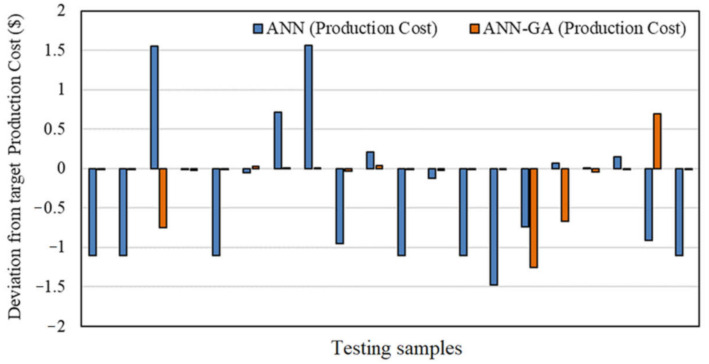
Deviation from the target values for the hybrid ANN-GA and single ANN methods.

Figure 13 presents the deviation from target values to compare the ANN and ANN-GA. These figures contain the relative deviation error values for testing data in two categories—single ANN and hybrid ANN-GA. The horizontal 0 line refers to target values, and columns refer to relative deviations for each predicted testing data from the target values. As is clear for all three variables, a single ANN provided a higher deviation from target values compared with the hybrid ANN-GA method. These observations show that hybrid methods offer higher accuracy and lower error compared with single methods.

### 3.5. Numerical Optimization

**Figure 14:**
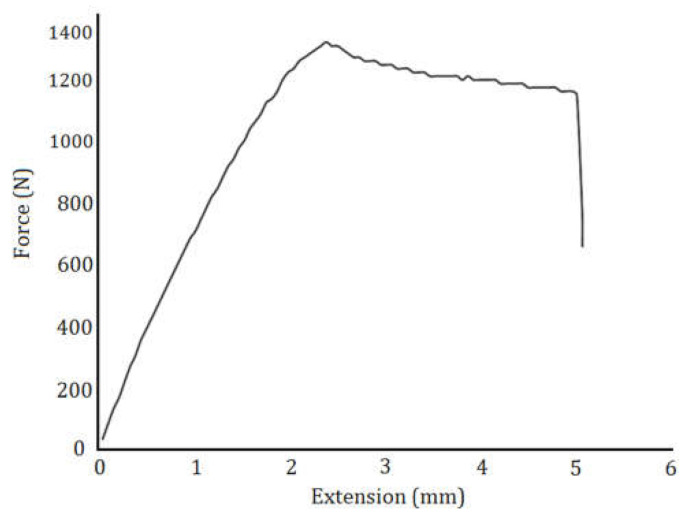
Extension–force diagram of the optimized specimen.

**Figure 15:**
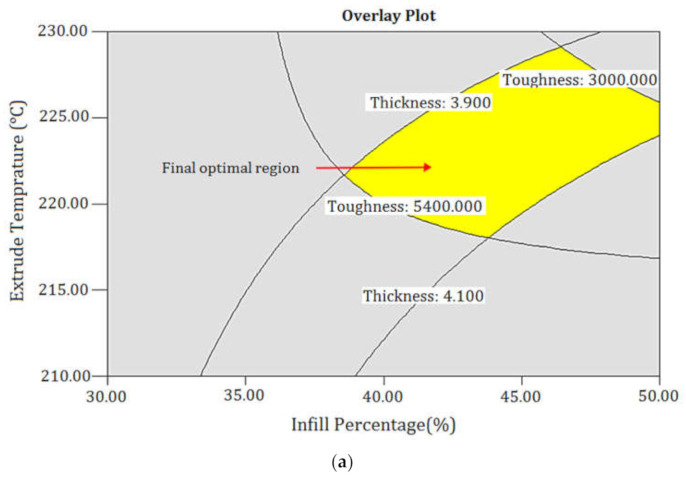

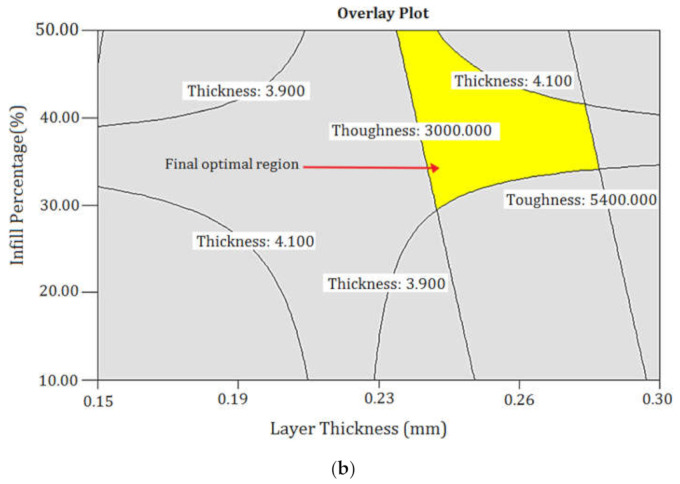
Overlaying contour maps in terms of (**a**) ET and IP and (**b**) IP and LT.

The process parameters were optimized based on a criterion defined in Table 11. The standard aimed to increase the toughness, achieve the desired thickness, and decrease the production cost of 3D printed parts. It was anticipated that the optimized specimen would demonstrate tough behavior at the least-possible production cost with the desired part thickness. The predicted and the experimental results for the implementation of the optimized process parameters are shown in Table 12. The optimum solution had a high level of desirability. Figure 14 depicts the force–extension graph of the tensile test specimens fabricated by the optimal setting. The optimized specimen’s improved toughness was more due to an increase in ductility rather than the specimen’s strength. By overlaying contour maps from multiple responses, RSM can be used to find the ideal window of operability. The overlaying contour maps to create ideal printed samples is shown in Figure 15. In each contour map, regions that did not meet the significations are grayed-out [32].

## 4. Conclusions

The present work aimed to enhance the production of PLA printed parts via an investigation of the toughness, thickness, and production cost of the tensile test specimens. Additionally, training was performed by the ANN and ANN-GA techniques for developing an accurate model for the prediction of toughness, part thickness, and production cost. This method was performed by MATLAB software and calculated a superb prediction of output parameters. The tensile test of samples not only provides a deep insight into the main PLA’s features, but can also present brilliant results of printed samples that are printed by some criteria such as IP, ET, and LT. The DOE of this study redcued125 tests to only 20 tests, which has a big impact on saving time and production cost. From the results obtained, the following concrete conclusions can be made. Although PLA is brittle in nature, the results confirm that it is feasible to improve the toughness of the printed parts to develop PLA’s end-use mechanical applications. Furthermore, as build time plays a major role in determining production cost, it is possible to reduce production cost without a significant impact on the desired properties. It can also be concluded that interaction between LT and IP is the main parameter that has an impact on the thickness of the printed part. It can be conceived that due to little shrinkage of the PLA, extruder temperature has less influence on the dimensional accuracy of the PLA. In addition, the optimized setting to enhance the producibility of PLA printed parts was a layer thickness of 0.28 mm, infill percentage of 34%, and extruder temperature of 222 °C. The improved toughness of the optimized specimen was due more to an increase in ductility rather than the strength of the specimen. The results also showed that a single ANN model could provide a higher deviation from the target values for all three outputs compared with the hybrid ANN-GA method. For future research, comparative analysis of the hybrid, ensemble, and deep learning models is strongly encouraged to improve the accuracy of the models. The research was accomplished under the constraints of PLA compatibility with existing fused filament fabrication installation, in the absence of the functional assistance of the machine. Although the mechanical properties and dimensional accuracy of PLA have already been studied, there is little literature on the toughness of the printed PLA with a honeycomb internal fill pattern.

## Figures and Tables

**Table 1:** Experimental data for the levels of independent factors.

Factor	Unit	Levels				
		−2	−1	0	1	2
LT	mm	0.1	0.15	0.2	0.25	0.3
IP	%	10	20	30	40	50
ET	C	190	200	210	220	230

**Table 2:** Experimental data for design of experiments (DOE) method.

Run	Input Factors			Output Responses			Type of Fracture
	LT	IP	ET	Toughness (N-mm)	Part Thickness (mm)	Production Cost ($)	
1	0.20	30.00	210.00	1829.27	3.98	17.73	Brittle
2	0.20	30.00	210.00	1394.35	3.84	17.73	Brittle
3	0.15	40.00	220.00	1157.86	3.88	21.72	Brittle
4	0.30	30.00	210.00	5164.36	3.68	13.77	Tough
5	0.20	30.00	210.00	1674.03	4.02	17.73	Brittle
6	0.25	40.00	200.00	5144.17	4.00	15.76	Tough
7	0.25	20.00	200.00	1835.62	3.82	15.25	Brittle
8	0.15	20.00	220.00	2239.94	4.48	20.2	Brittle
9	0.20	30.00	210.00	4112.96	4.04	17.73	Tough
10	0.15	40.00	200.00	1520.79	3.98	21.72	Brittle
11	0.20	30.00	210.00	1140.16	4.08	17.73	Brittle
12	0.20	10.00	210.00	1167.21	3.86	16.72	Brittle
13	0.10	30.00	210.00	830.976	3.98	27.19	Brittle
14	0.15	20.00	200.00	817.052	4.08	20.2	Brittle
15	0.20	30.00	230.00	2644.34	4.08	17.23	Brittle
16	0.20	30.00	190.00	2075.45	3.74	17.23	Brittle
17	0.20	50.00	210.00	2462.57	3.9	18.25	Brittle
18	0.25	40.00	220.00	4489.05	4.12	15.76	Tough
19	0.25	20.00	220.00	5046.5	3.8	15.25	Tough
20	0.20	30.00	210.00	1393.06	3.86	17.73	Brittle

**Table 3:** Model evaluation metrics.

Accuracy and Performance Index	Description
*Correlation coefficient =* $\frac{N\sum_{\;}^{\;}{\left(AP\right)}^{\;}-\sum_{\;}^{\;}{\left(A\right)}^{\;}\sum_{\;}^{\;}{\left(P\right)}^{\;}}{\sqrt{[N\sum_{\;}^{\;}A^{2}-{\left(\sum_{\;}^{\;}A\right)}^{\;}{}^{2}]{\left[N\sum_{\;}^{\;}P^{2}-{\left(\sum_{\;}^{\;}AP\right)}^{\;}{}^{2}\right]}^{\;}{}^{\;}}}$	– *N* is the number of data – *A* is the desired output value and *P* is the output value.
*RMSE =* $\sqrt{\frac{1}{N}\overset{\;}{\underset{\;}{\sum }}{\left(A-P\right)}^{2}}$	

**Table 4:** Material properties of the data sheet.

Property	Value
Full name	Polylactic acid (PLA)
Melting point	150 to 160 °C (302 to 320 °F)
Glass transition	60–65 °C
Injection mold temperature	178 to 240 °C (353 to 464 °F)
Density	1.210–1.430 g·cm^−3^
Chemical formula	(C_3_H_4_O_2_)n
Crystallinity	37%
Tensile modulus	2.7–16 GPa
molecular weight (Mw)	112 kg/mol ± 1733
Polydispersity (M_W_/M_N_)	1.65 ± 0.05

**Table 5:** Fused filament fabrication (FFF) build parameters.

No	Build Parameters	Unit	Value
1	Nozzle diameter	mm	0.45
2	Extrusion width	mm	0.45
3	Top solid layer	-	6
4	Bottom solid layers	-	6
5	Default printing speed	mm/min	3600
6	Retraction speed	mm/min	1800
7	Outline overlap	-	Full honeycomb
8	Interior fill percentage	%	15

**Table 6:** Analysis of variance (ANOVA) for toughness.

Source	Sum of Squares (SOS)	Df	Mean Square (MS)	F-Value (F-v)	*P*-Value (*P*-v)
Model	1.694 × 10^−3^	4	4.235 × 10^−4^	13.04	<0.0001
LT	1.228 × 10^−3^	1	1.228 × 10^−3^	37.81	<0.0001
IP	1.250 × 10^−4^	1	1.250 × 10^−4^	3.85	0.0687
ET	8.980 × 10^−5^	1	8.980 × 10^−5^	2.76	0.1171
(IP) × (ET)	2.513 × 10^−4^	1	2.513 × 10^−4^	7.74	0.0140
Residual	4.872 × 10^−4^	15	3.248 × 10^−5^		
Lack of Fit (LOF)	1.747 × 10^−4^	10	1.747 × 10^−5^	0.28	0.9591
Pure Error (PR)	3.125 × 10^−4^	5	6.250 × 10^−5^		
Cor Total (CT)	2.181 × 10^−3^	19			
Pred R-Square	0.6747	Adj R-Squared	0.7171	R-Squared	0.7766

**Table 7:** ANOVA for thickness.

Source	SOS	Df	MS	F-v	*P*-v
Model	0.89	6	0.15	4.46	0.0115
LT	0.20	1	0.20	5.98	0.0294
IP	0.024	1	0.024	0.73	0.4096
E)	0.15	1	0.15	4.48	0.0542
(LT) × (IP)	0.36	1	0.36	10.92	0.0057
(LT) × (ET)	0.061	1	0.061	1.85	0.1968
(IP) × (ET)	0.092	1	0.092	2.79	0.1185
Residual	0.43	13	0.033		
PR	0.049	5	9720 × 10^−3^		
LOF	0.38	8	0.048	4.91	0.0482
CT	1.32	19			
Pred R-Square	−0.5694	Adj R-Squared	0.5220	R-Squared	0.6730

**Table 8:** ANOVA for production cost.

Source	SOS	Df	MS	F-v	*P*-v
Model	6.769 × 10^−5^	5	1.354 × 10^−5^	1464.91	<0.0001
LT	6.592 × 10^−5^	1	6.592 × 10^−5^	7133.12	<0.0001
IP	1.555 × 10^−6^	1	1.555 × 10^−6^	168.23	<0.0001
ET	0.000	1	0.000	0.000	1.0000
IP^2^	4.940 × 10^−8^	1	4.940 × 10^−8^	5.35	0.0365
ET^2^	1.927 × 10^−7^	1	1.927 × 10^−7^	20.85	0.0004
PE	0.000	5	0.000		
LOF	1.294 × 10^−7^	9	1.438 × 10^−8^		
Residual	1.294 × 10^−7^	14	9.241 × 10^−9^		
CT	6.782 × 10^−5^	19			
Pred R-Square	0.9940	Adj R-Squared	0.9974	R-Squared	0.9981

**Table 9:** Results for the training phase.

Output Factor	ANN	Correlation Coefficient	RMSE	ANN-GA		Correlation Coefficient	RMSE
	No. of Neurons			Pop. Size	Max Gen.		
Toughness (N-mm)	10	0.7877	924.5529274	50	320	0.9439	633.6373621
	12	0.8782	908.0737946	100	210	0.8692	734.6853877
	14	0.7964	893.2048644	150	360	0.9642	453.8843405
	16	0.8789	694.1594251	200	110	0.9186	654.6824998
Part thickness (mm)	10	0.7671	0.12949	50	320	0.9362	0.045035408
	12	0.8788	0.075333178	100	210	0.93	0.042059003
	14	0.5173	0.084915266	150	360	0.7768	0.059754932
	16	0.6324	0.099691882	200	110	0.8538	0.077821436
Production cost ($)	10	0.8531	1.960663	50	320	09485	1.288136157
	12	0.9636	0.970732923	100	210	0.8956	1.556494043
	14	0.8235	3.830106928	150	360	0.9754	1.011613758
	16	0.842	2.267871729	200	110	0.9105	1.29979527

**Table 10:** Results for the testing phase.

Output Factor	ANN	Correlation Coefficient	RMSE	ANN-GA		Correlation Coefficient	RMSE
	No. of Neurons			Pop. Size	Max Gen.		
Toughness (N-mm)	12	0.91	651.7539629	150	360	0.9791	277.4633823
Part thickness (mm)		0.8911	0.118439425			0.9904	0.036062371
Production Cost ($)		0.938	0.861473905			0.9762	0.569953845

**Table 11:** Criteria, effective inputs, and responses of each parameter.

Responses/Parameters		Name	Goal	Lower Limit	Upper Limit	Lower Weight	Upper Weight	Importance
Parameters		LT	Is in range	0.1	0.3	1	1	-
		IP	Is in range	10	50	1	1	-
		ET	Is in range	190	230	1	1	-
Responses	Criteria	Toughness	Maximum	817	5500	1	1	1
		Thickness	is goal = 4	3.68	4.98	1	1	1
		Cost	Minimum	13.77	27.19	1	1	1

**Table 12:** Experimental validation and predicted optimum outputs.

Sol.	Optimum Inputs			Desirability		Output Responses		
	LT	IP	ET			Toughness (N-mm)	Thickness (mm)	Production Cost ($)
1	0.28	38	222	0.99	Actual	5097.727	3.72	14.77
					Predicted	5399.99	4.000	14.372
					Error%	−5.93%	−7.5%	2.23%

## Data Availability

Data sharing not applicable.

## References

[1] Qattawi A., Alrawi B., Guzman A. (2017). Experimental optimization of fused deposition modelling processing parameters: A design-for-manufacturing approach. Procedia Manuf..

[2] Sajan N., John T., Sivadasan M., Singh N. (2018). An investigation on circularity error of components processed on Fused Deposition Modeling (FDM). Mater. Today.

[3] Sood A.K., Ohdar R., Mahapatra S.S. (2009). Improving dimensional accuracy of fused deposition modelling processed part using grey Taguchi method. Mater. Des..

[4] Liu X., Zhang M., Li S., Si L., Peng J., Hu Y. (2017). Mechanical property parametric appraisal of fused deposition modeling parts based on the gray Taguchi method. Int. J. Adv. Manuf. Technol..

[5] Dong G., Wijaya G., Tang Y., Zhao Y.F. (2018). Optimizing process parameters of fused deposition modeling by Taguchi method for the fabrication of lattice structures. Addit. Manuf..

[6] Mahmood S., Qureshi A., Talamona D. (2018). Taguchi based process optimization for dimension and tolerance control for fused deposition modelling. Addit. Manuf..

[7] Rao R.V., Rai D.P. (2016). Optimization of fused deposition modeling process using teaching-learning-based optimization algorithm. Eng. Sci. Technol. Int. J..

[8] Ceretti E., Ginestra P., Neto P., Fiorentino A., Da Silva J. (2017). Multi-layered scaffolds production via Fused Deposition Modeling (FDM) using an open source 3D printer: Process parameters optimization for dimensional accuracy and design reproducibility. Procedia CIRP.

[9] Griffiths C., Howarth J., Rowbotham G.D.-A., Rees A. (2016). Effect of build parameters on processing efficiency and material performance in fused deposition modelling. Procedia CIRP.

[10] Lieneke T., Denzer V., Adam G.A., Zimmer D. (2016). Dimensional tolerances for additive manufacturing: Experimental investigation for Fused Deposition Modeling. Procedia CIRP.

[11] Rezaie R., Badrossamay M., Ghaie A., Moosavi H. (2013). Topology optimization for fused deposition modeling process. Procedia CIRP.

[12] Ivanova O., Williams C., Campbell T. (2013). Additive manufacturing (AM) and nanotechnology: Promises and challenges. Rapid Prototyp. J..

[13] Buys Y., Aznan A., Anuar H. (2018). Mechanical properties, morphology, and hydrolytic degradation behavior of polylactic acid/natural rubber blends. Proceedings of the IOP Conference Series: Materials Science and Engineering (ICAMME 2017).

[14] Yadav D., Chhabra D., Gupta R.K., Phogat A., Ahlawat A. (2020). Modeling and analysis of significant process parameters of FDM 3D printer using ANFIS. Mater. Today.

[15] Ali F., Chowdary B.V. (2019). Natural Frequency prediction of FDM manufactured parts using ANN approach. IFAC Pap..

[16] Sheoran A.J., Kumar H. (2020). Fused Deposition modeling process parameters optimization and effect on mechanical properties and part quality: Review and reflection on present research. Mater. Today.

[17] Moradi M., KaramiMoghadam M. (2019). High power diode laser surface hardening of AISI 4130; statistical modelling and optimization. Opt. Laser Technol..

[18] Moradi M., Karami Moghadam M., Shamsborhan M., Bodaghi M., Falavandi H. (2020). Post-Processing of FDM 3D-Printed Polylactic Acid Parts by Laser Beam Cutting. Polymers.

[19] Azadi M., Azadi S., Zahedi F., Moradi M. Multidisciplinary optimization of a car component under NVH and weight constraints using RSM. Proceedings of the ASME 2009 International Mechanical Engineering Congress and Exposition.

[20] Plymill A., Minneci R., Greeley D.A., Gritton J. (2016). Graphene and Carbon Nanotube PLA Composite Feedstock Development for Fused Deposition Modeling. Ph.D. Thesis.

[21] Benyounis K.Y., Olabi A.G., Hashmi M.S.J. (2009). Mechanical properties, weld bead and cost universal approach for CO_2_ laser welding process optimisation. Int. J. Comput. Mater. Sci. Surf. Eng..

[22] Moradi M., Meiabadi S., Kaplan A. (2019). 3D printed parts with honeycomb internal pattern by fused deposition modelling; experimental characterization and production optimization. Met. Mater. Int..

[23] Sideratos G., Ikonomopoulos A., Hatziargyriou N.D. (2020). A novel fuzzy-based ensemble model for load forecasting using hybrid deep neural networks. Electr. Power Syst. Res..

[24] Robinson M.C., Glen R.C. (2020). Validating the validation: Reanalyzing a large-scale comparison of deep learning and machine learning models for bioactivity prediction. J. Comput. Aided Mol. Des..

[25] Amid S., Gundoshmian T.M. (2017). Prediction of output energies for broiler production using linear regression, ANN (MLP, RBF), and ANFIS models. Environ. Prog. Sustain. Energy.

[26] Chen G., Shen Z., Iyer A., Ghumman U.F., Tang S., Bi J., Chen W., Li Y. (2020). Machine-Learning-Assisted De Novo Design of Organic Molecules and Polymers: Opportunities and Challenges. Polymers.

[27] Huang L., Ling C. (2020). Practicing deep learning in materials science: An evaluation for predicting the formation energies. J. Appl. Phys..

[28] Mellit A. (2010). ANN-based GA for generating the sizing curve of stand-alone photovoltaic systems. Adv. Eng. Softw..

[29] Elbadawi M., Castro B.M., Gavins F.K., Ong J.J., Gaisford S., Pérez G., Basit A.W., Cabalar P., Goyanes A. (2020). M3DISEEN: A novel machine learning approach for predicting the 3D printability of medicines. Int. J. Pharm..

[30] Hu C., Hau W.N.J., Chen W., Qin Q.H. (2020). The fabrication of long carbon fiber reinforced polylactic acid composites via fused deposition modelling: Experimental analysis and machine learning. J. Compos. Mater..

[31] Torres J., Cotelo J., Karl J., Gordon A.P. (2015). Mechanical property optimization of FDM PLA in shear with multiple objectives. JOM.

[32] Moradi M., Karami Moghadam M., Shamsborhan M., Bodaghi M. (2020). The synergic effects of FDM 3D printing parameters on mechanical behaviors of bronze poly lactic acid composites. J. Compos. Sci..

